# Plant-Derived Smoke and Karrikin 1 in Seed Priming and Seed Biotechnology

**DOI:** 10.3390/plants12122378

**Published:** 2023-06-19

**Authors:** Jan Kępczyński, Ewa Kępczyńska

**Affiliations:** Institute of Biology, University of Szczecin, Waska 13, 71-415 Szczecin, Poland; ewa.kepczynska@usz.edu.pl

**Keywords:** smoke-priming, somatic embryogenesis, smoke water, karrikin, KAR_1_-priming

## Abstract

Plant-derived smoke and smoke water (SW) can stimulate seed germination in numerous plants from fire-prone and fire-free areas, including cultivated plants and agricultural weeds. Smoke contains thousands of compounds; only several stimulants and inhibitors have been isolated from smoke. Among the six karrikins present in smoke, karrikin 1 (KAR_1_) seems to be key for the stimulating effect of smoke. The discovery and activity of highly diluted SW and KAR_1_ at extremely low concentrations (even at ca. 10^−9^ M) inducing seed germination of a wide array of horticultural and agricultural plants have created tremendous opportunities for the use of these factors in pre-sowing seed treatment through smoke- or KAR_1_-priming. This review presents examples of effects exerted by the two types of priming on seed germination and seedling emergence, growth, and development, as well as on the content of some compounds and enzyme activity. Seed biotechnology may involve both SW and KAR_1_. Some examples demonstrate that SW and/or KAR_1_ increased the efficiency of somatic embryogenesis, somatic embryo germination and conversion to plantlets. It is also possible to stimulate in vitro seed germination by SW, which allows to use in orchid propagation.

## 1. Introduction

**Figure 1:**
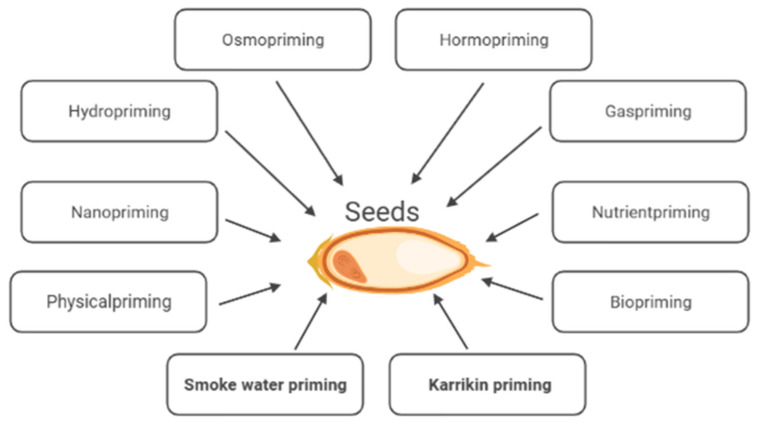
Methods of seed priming.

After they have been sown, seeds are exposed to various environmental factors influencing seed germination and seedling establishment. These processes seldom take place under optimal conditions. Adverse environmental factors, e.g., extreme temperatures, drought, or high salinity, may slow down germination, prevent uniform germination, and/or cause a low percentage of germination, poor seedling emergence, and irregular plant development, which consequently leads to a reduction in the quality and size of the yield [1,2]. Seed priming technology is an economically successful, viable key strategy to increase crop production under non-stressful and stressful environmental conditions. Conventional seed priming, e.g., hydropriming, osmopriming and matriconditioning, involves seed hydration, allowing metabolism to proceed but preventing radicle protrusion. So far, various priming methods, e.g., hormopriming, gas priming, physical priming, biopriming etc., responsible for the induction of various physio-biochemical traits improving seed vigor, have been developed [2,3] (Figure 1).

Since crop cultivation under adverse environmental conditions may reduce yields by up to 50% [4], various cost-effective and easily applicable types of priming should be more widely used, and the search for new pre-sowing techniques should be developed. Demonstrating the ability to stimulate seed germination and seedling growth in many plant crops by plant-derived smoke and smoke-derived KAR_1_ has generated new opportunities for their use in the pre-sowing treatment of seeds by SW- and KAR_1_-priming. This review also focuses on applying SW and/or KAR_1_ in plants in vitro cultures, the latter being a basis of plant biotechnology, to regulate somatic embryogenesis and seed germination in mass plant propagation. 

## 2. The Importance of Discovering the Biological Activity of Smoke and Compounds Isolated from It

Having observed that vegetation regenerates after a fire, farmers began to use smoke to treat seeds. In many areas, farmers have used fire and plant-derived smoke to treat seeds to stimulate their germination [5]. Initial data on the effect of smoke on seed germination were published by De Lange and Boucher in 1990 [6]. To those scientists, we owe the discovery of smoke-saturated water (SW) being as effective as smoke alone. This information generated great interest, followed by a large number of publications on the influence of smoke and SW on seed germination in several plant species. The role of smoke in seed germination was also described in reviews [5,7,8,9,10,11,12]. It has been reported that seeds or seedlings of over 1300 plant species respond to smoke or SW [7,12]. Smoke/SW stimulate seed germination in plants derived from areas where fires can occur and crops, vegetables, and weeds [5,9]. Very interesting was the demonstration that SW can be prepared by bubbling, through water, when burning leaves of monocotyledons, dicotyledons and gymnosperms leaves of plants originating not only from fire-prone areas, and also that SW does not lose its activity even after several years of storage [13]. Results of experiments on the beneficial effect of smoke on seed germination and seedling growth were very quickly put into practice by the development of various commercial preparations [14]. 

**Figure 2:**
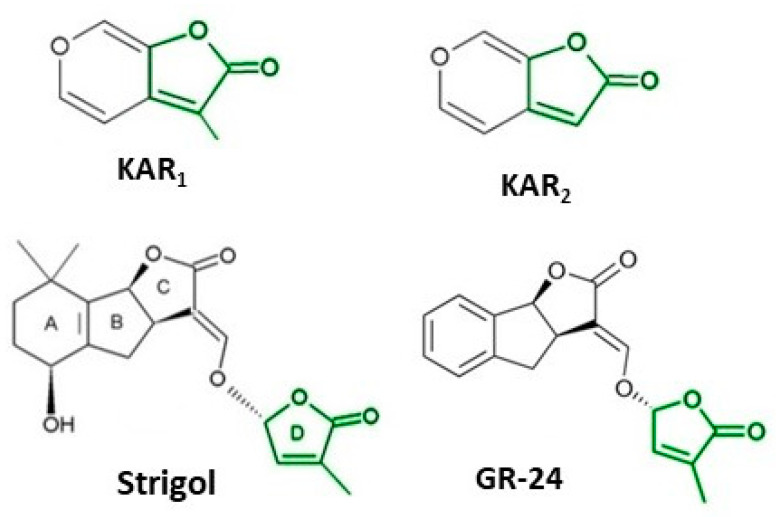
The structure of some bioactive karrikins and strigolactones. KAR_1_ - 3 methyl-2*H*-furo[2,3-c]pyran-2-one, most often the most active of the karrikins and found in smoke at the highest concentration; KAR_2_-karrikin with activity towards Arabidopsis seeds higher than that of KAR_1_; strigol-natural strigolactone; GR24-synthetic strigolactone. Both karrikins and strigolactones have a butenolide ring. Karrikins and GR24 have similar effects on seed germination, e.g., Arabidopsis and *Brassica tournefortii*, seedling photomorphogenesis and expression of genes [17,18]. KAR_1,_ in contrast to GR24, could not stimulate the germination of parasitic weed seeds.

It took many years to search, among thousands of compounds in smoke, for the compound responsible for stimulating smoke-driven germination. The search proved successful only in 2004 when two independent teams isolated butenolide, currently called karrikinolide or karrikin 1 (KAR_1_), from plant-derived smoke [15] and from burnt cellulose [16] (Figure 2). 

Smoke is now known to contain also other karrikins, numbered from KAR_1_ to KAR_6_ [8]. KAR_1_ is usually the most active of all known karrikins; it is present in the highest concentration of smoke, 5.5 to 38 times higher than other karrikins [8]. The KAR_1_ concentration of 6.7 × 10^−7^ M was found to correspond to a SW dilution of 1:100 [7]. The compound has been found to stimulate seed germination of plants from fire-prone areas and fire-free regions; the germination of crops and weeds is also stimulated. KAR_1_ was shown to be very active at extremely low concentrations such as 10^−10^–10^−7^ M. Various times of imbibition in a KAR_1_ solution, e.g., from 1 min for *Emmenanthe penduliflora* seeds [7] to 6 h for *Avena fatua* caryopses [19] was sufficient to stimulate germination. KAR_1_ can be synthesized using different substrates and is commercially available (Table 1).

Glyceronitrile was reported to appear after fire; in addition to karrikins, ethylene and nitric oxide (NO) are also present in smoke and induce dormancy release in many seeds. They all are recognized as signals in upper soil layers, characteristic of fire and smoke, for dormant seeds to germinate [25] (Table 2). 

Seed germination is stimulated by hydroquinone, and seedling growth is improved by catechol, both compounds being detected in smoke as well [30]. Further research has shown that there are seeds which respond to KAR_1_, but their germination is neither affected nor stimulated by smoke [9]. Moreover, weak—as opposed to strong—SW dilutions inhibit seed germination. Such an effect of SW has been suspected to be related to the presence in the smoke of certain inhibitors antagonistic to karrikins and/or other stimulants. Other experiments revealed the presence of three compounds: 3,4,5-trimethyl furan-2 (5*H*)-one (TMB), 5,5-dimethylfuran-2(5*H*)-one and (5RS)-5-ethylfuran-2(5*H*)-one that inhibited seed germination and exerted an antagonistic effect towards that of KAR_1_ [28,31]. Thus, the effect of smoke should be considered as a comprehensive biological response to the interacting compounds present in the smoke. Some studies showed TMB to be present in the soil in concentrations higher than those of KAR_1_ and to be more water-soluble than KAR_1_ [12]. It has been proposed that TMB accumulated in the soil is diluted and washed away by heavy rainfall, which makes it possible for the stimulatory effect of KAR_1_ to be expressed. This agrees with a previous suggestion that smoke plays a dual function in regulating seed germination in soil [32]. 

## 3. Seed Priming with SW or KAR_1_ Solution

Since the high effectiveness of SW at high dilutions and KAR_1_ at low concentrations as stimulants of seed germination and/or seedling development have been demonstrated [8,9,11,33], the agents have become a focus of research on seed priming, a pre-sowing technique. For priming, seeds were imbibed in Petri dishes on filter paper moistened with appropriate solutions of SW or KAR_1_ or were soaked in solutions of these agents (Table 3).

Then, the SW- or KAR_1_-primed seeds were surface dried or dried to air dry level and sown immediately or after storage in Petri dishes or soil. Subsequently, germination, seedling emergence, seedling growth and/or certain indicators of metabolism were determined. SW- or KAR_1_-priming were applied to seeds of natural plants in the revegetation strategy and to improve seedling emergence and the development of cultivated plants. Early experiments with *Themeda triandra*, a dominant fire-climax grass, showed seeds imbibed in an SW solution and then dried and stored at 25 °C to germinate better than untreated seeds [45]. The cited authors rightly concluded that such seed pre-treatment could be used to revegetate the plant. Later, other promising results were obtained from research on SW application to prime seeds of cultivated plants. Germination of *Solanum centrale*, *S. dioicum* and *S. orbiculatum* seeds, vigor index and 18-d old seedling length were found to increase when the seeds were imbibed in SW and immediately transferred to Petri dishes with agar [44]. However, it is not known whether the stimulating effect of SW-priming would also persist after seed drying and storage. In another experiment, soaking of *Silybum marianum* seeds, a plant native to Europe, Asia, and Africa, at present cultivated for the pharmaceutical industry [43] in SW, despite drying, enhanced the germination speed at 25 °C, vigor index and 14-days old seedling length. Soaking *Dactylis glomerata* seeds in SW solution and drying them before sowing in the field increased germination, seedling emergence under field conditions and biomass after ten weeks of crop cultivation [48]. That beneficial effects can also appear under environmental conditions was an important information. In another experiment, soaking of *Zea mays* seeds in SW increased the rate of seed germination and fresh weight of shoots and roots in 8-day-old seedlings [46]. Moreover, chlorophyll and carotenoid contents were higher in seedlings obtained from the primed seeds.

Because SW-priming positively affected seed germination, seedling emergence and/or seedling development, experiments were carried out to explore the effects of KAR_1_-priming or both SW- and KAR_1_-priming, in one of the experiments, the sowing of *Zea mays* kernels soaked in an SW or KAR_1_ solution, followed by surface drying, increased the plant height, root and shoot weight and dry weight after 30 days of greenhouse cultivation [47], indicating the advantages of both SW- and KAR_1_-priming. However, whether this positive effect persisted until the end of plant development and whether it was reflected in the yield is unknown. Doubtless, the use of primed seeds in commercial practice would be enhanced if positive effects were not reduced due to the post-treatment seed drying and drying and storage. Therefore, it was essential to find out if seeds subjected to KAR_1_-priming could be stored for some time without losing the beneficial priming effect. In one experiment, the effect of priming on the development of plants after cultivation under environmental conditions was addressed using *Daucus carota*. SW and KAR_1_ were applied during the soaking of *D. carota* seeds, air-dried before sowing, increased germination in soil and seedling growth after 120 days of growth under natural conditions [39]. Moreover, both priming techniques increased the photosynthesis rate and the contents of carotene and ascorbic acid. *Capsicum annuum* seeds, primed with KAR_1_ by soaking, showed improved germination of both immature and mature seeds. The technique worked better in immature prime than hydropriming [35]. Likewise, an advantageous effect of KAR_1_-priming was observed in 20-day-old *C. annuum* seedlings when the fresh weight of the seedlings grown on a peat moss medium was determined. The stimulatory effect of KAR_1_ also included an increase in catalase (CAT), ascorbate peroxidase (APX) and superoxide dismutase (SOD) activities in both immature and mature seeds.

So far, various priming technologies have been successfully used to enhance resistance to abiotic stresses, consistently improving overall plant growth [1]. SW and KAR_1_ have been demonstrated to have a positive effect not only on seed germination and seedling development under optimal conditions but also under stress [11], so it was logical to conduct research aimed at finding out whether SW- and/or KAR_1_-priming can be used to increase plant tolerance to various abiotic stresses. However, only limited, albeit important, information has been obtained so far through experiments focusing on using both above-priming techniques to improve seed germination, seedling emergence and establishment under various stress conditions. Extreme temperatures, too low or too high, are known to have an adverse impact on seed germination and plant development by disrupting metabolism, damaging structures, and ultimately leading to the death of cells, organ tissues and even the entire organism. Priming *Lycopersicon esculentum* by soaking the seeds in a KAR_1_ solution increased the seedling vigor after 7-day incubation at various temperatures, including sub- and supraoptimal [41].

Moreover, KAR_1_-priming enhanced seedling vigor under salt and osmotic stress—the cultivation of *Brassica napus* cv. English giant, an annual herbaceous leafy vegetable, can be limited due to the high temperature and water stress. SW-priming and, to a larger extent, KAR_1_-priming increased seed germination at heat stress (40 °C) [34]. 

Likewise, SW- and KAR_1_-priming by imbibing seeds of *Eragrostis tef*, a major cereal crop in the Horn of Africa countries, were used to test germination and vigor of 7-day-old seedlings at various temperatures, including 40 °C, and at osmotic stress [40]. The treatment improved both germination and seedling vigor. Similarly, SW-priming of *Oryza sativa* seeds increased seed germination and seedling vigor under salt stress simulated by NaCl solutions [42]. Effects of priming included an increase in the chlorophyll and carotenoid contents. In addition, priming increased the contents of K^+^ and Ca^+^, but decreased that of Na^+^. In *Ceratotheca triloba*, SW- and KAR_1_-priming increased seed germination after 25 days under osmotic stress caused by PEG 6000 solutions [36]. Both priming techniques also improved the growth of 25-day-old seedlings under stress. The effect of KAR_1_-priming on the alleviation of stress caused by cadmium, a pollutant prevalent in arable lands, was studied on seeds of *Coriandrum sativum*, an important herbaceous plant cultivated in various regions of the world and used in pharmacy, cosmetology, and various cuisines as a spice [37]. Cadmium was found to inhibit seed germination and to reduce the fresh and dry weight of 15-day-old seedlings. The inhibitory effect declined when primed seeds were used. The beneficial effect of priming on cadmium stress tolerance was associated with increased leaf osmotic potential, membrane stability, photosynthesis rate and proline content. Moreover, KAR_1_-priming resulted in decreased contents of malondialdehyde (MDA) and hydrogen peroxide (H_2_O_2_) and electrolyte leakage. In contrast, antioxidant enzymes: superoxide dismutase (SOD), peroxidase (POD) and catalase (CAT) in seedlings were found to enhance their activity. The effect of KAR1-priming of Cucumis melo seeds sown, after surface drying, in peat moss at two depths and kept at 20 and 25 °C in the controlled climatic room for 24 days, on seedling emergence and seedling fresh and dry weight was also examined [38]. The KAR1-priming advantage was particularly evident if the seeds were of poorer quality and were sown too deep at a sub-optimal temperature of 20 °C. KAR1-priming was observed to increase seedling emergence, speed, and final percentage, and seedling fresh weight at 20 °C more effectively than at 25 °C when the seeds were sown deeper. 

It is probably still long before SW and/or KAR_1_ will be commercially applied in priming technology. One can only consider the potential use of SW- and KAR_1_-priming in increasing seed tolerance to adverse environmental factors. So far, research has been limited to assessing the effects on germination and seedling development, often on Petri dishes. The impact of both types of priming on plant development and yield under environmental conditions in most cases has not been determined. In the experiments, SW- or KAR_1_-primed seeds were most often used immediately after the treatment or only after surface drying, and it is known that other priming techniques and KAR_1_-priming diminish the seed priming benefit as a result of drying. Perhaps a solution would be to dry the seeds gradually, similarly to the drying manner recommended for somatic embryos.

Considering the practical application of SW or KAR_1_ in priming, if the seeds are sensitive to SW, SW-priming seems more valuable because the SW preparation is easy and cost-free. Although KAR_1_ is expensive due to its effectiveness at very low concentrations, its use is also promising. Knowledge of KAR_1_-primed seed metabolism is still scant. Application of SW and/or KAR_1_ in combination with other conditioning methods, e.g., osmopriming or matripriming, to seeds of economically important plants requires further research. 

## 4. SW and KAR_1_ in Seed Biotechnology

The use of both SW and KAR_1_ in vitro cultures of various plant materials has been a focus of several studies. As fire and smoke were found to be capable of inducing flowering in some plants, e.g., *Cyrtanthus ventricosus* and *Watsonia borbonica* [49,50], experiments were conducted to follow the response of pollen of various plants from fire-prone areas. SW or KAR_1_ were demonstrated to stimulate pollen germination and tube growth of various plant species; therefore, it was correctly concluded that both agents could increase flower pollination, leading to increased fertilization and seed yield [51,52]. However, no data are showing the effect on the seed yield. It was very interesting and extremely important to discover that, under natural conditions and in vitro, it is possible to generate embryos from somatic cells without fertilization. Somatic embryogenesis, which makes it possible to produce artificial seeds, is of great interest to both the researchers striving to explain the process and the practitioners who could use the process for the mass multiplication of plants. Application of somatic embryogenesis in practice requires optimalisation of the process for the sake of high efficiency, particularly with respect to cotyledonary embryos, the most advanced stage of embryo development. Experiments were carried out to examine the suitability of SW and KAR_1_ in improving somatic embryogenesis (Table 4). 

However, only a few examples of SW or KAR_1_ are being applied to improve somatic embryogenesis. SW was shown to increase embryogenesis efficiency in *Pelargonium hortorum* [54] markedly. Important insights were gained, including the observation that SW increased the formation of cotyledonary embryos. When SW was applied to the hypocotyl explant or during the induction phase, the number of cotyledonary embryos increased fivefold. In *Pinus wallichiana*, SW used in the induction medium increased the number of cotyledonary embryos by about 3.5 [55]. SW was also found to increase the occurrence of secondary embryogenesis in *Brassica napus* [57]. These data may suggest that karrikin in the smoke was likely responsible for its stimulatory effect. It is important to note that KAR_1_ accelerated the development of torpedo embryos in *Baloskion tetraphyllum* [53]. Knowledge of the effect of SW and KAR_1_ on somatic embryo germination and conversion to plantlets is incomplete. An experiment with *P. wallichiana* showed SW to stimulate the germination of cotyledonary somatic embryos and to increase the number of surviving seedlings [55]. Likewise, KAR_1_ improved both somatic embryo germination and plantlet development in *B. tetraphyllum* [53]. Since SW and KAR_1_ were initially known as inducers of in vivo seed germination, it is understandable that they were applied to find out if they would be useful in inducing the in vitro seed germination. One of the methods of orchid production involves the sowing of seeds. Therefore, using agents that improve in vitro orchid seed germination is appropriate. SW stimulated the germination of asymbiotic seeds, protocorm differentiation and plant regeneration in *Vanda parviflora*, an epiphytic orchid [59]. Also, seed germination and plant recovery of the epiphytic orchid *Xenikophyton smeeanum* and *Tulbaghia ludwigiana*, a popular garden plant, was increased by SW [60].

Similarly, SW stimulated seed germination and the formation of protocorms in the epiphytic orchid *Ansellia africana* [58]. However, KAR_1_ could not induce germination or protocorm formation, which indicates that the SW activity can be associated with compounds other than karrikin(s) or other stimulant(s) present in SW. Seeds of different plant species, e.g., *Aloe arborescens*, were stimulated to germinate by SW [61]. Therefore, it has been demonstrated that SW could be used in orchid and aloe propagation. An in vitro experiment involving *Balsamorhiza deltoidea* and *B. sagittata*, plants from fire-prone areas of America, showed that, like in seeds of some plant species, KAR_2_—in contrast to—KAR_1_ was active in vivo as a stimulant [62].

Data on using both SW and KAR_1_ in seed biotechnology are promising, although too scant. There are no examples of KAR_1_ interaction with plant hormones in the regulation of somatic embryogenesis. The effects of the compound on the level of phytohormones are unknown, and the mechanism of in vitro treatments that considers the molecular level requires research.

## 5. Mechanism of Karrikin Signalling

**Figure 3:**
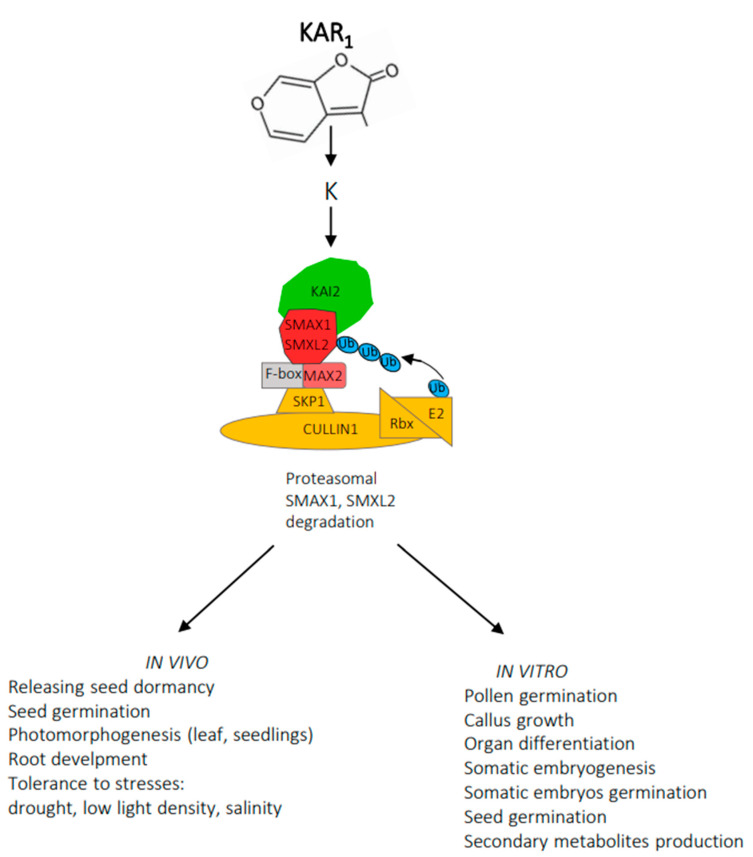
A proposed mechanism of KAR_1_ signalling. KAI2 (KARRIKIN INSENSITIVE2)-α,β hydrolases performing enzyme and receptor functions; K -a putative karrikin-derived molecule; F-box –recognizes substrate; Skp1 –substrate adaptor; Cullin –regulates complex activity; Rbx –facilitates transfer ubiquitin (Ub); E2 –transfers the ubiquitin to the substrate.

Recently, great progress in elucidating the karrikin signaling mechanism has been observed (Figure 3). 

Several studies indicate that KAR_1_ must be metabolized for an answer to this compound to emerge (63). KAR_1_ is metabolized to K (a putative karrikin-derived molecule). Activation of KAI2 (KARRIKIN INSENSITIVE2)-α,β hydrolases by K enables interaction with MAX2, an F-box protein part of Skp1-Cullin-F-box (SCF) E3 ubiquitin ligase complex. MAX2 recognizes SMAX1 (SUPPRESSOR OF MAX2), and SMXL2 (SMAX1-LIKE2), proteins that prevent the appearance of a response to KAR_1_. SMAX1 and SMXL2 proteins undergo polyubiquitination, followed by proteosomal degradation leading to the emergence of a response characteristic of KAR_1_ [63]. Fascinating is the great similarity in the signalling mechanism of both butenolide molecules, karrikins, unidentified in plants, and plant hormones, strigolactones [63,64]. MAX2 also mediates responses to strigolactones, which activate D14 (DWARF14)- α,β hydrolases structurally similar to KAI2 [18,65]. D14 acts with MAX2 to target SMXL6, SMXL7 and SMXL8 for ubiquitination and degradation. D14 can also target SMAX1 and SMXL2 when an adequate agonist is present. Moreover, there are also similarities in the signaling mechanism of karrikins and plant hormones such as auxins, jasmonates and gibberellins. All response systems involve the Skp1/Cullin/F-box-E3 ubiquitin ligase complex and the ubiquitination of the regulatory protein and its degradation by the 26S proteosome. Studies that involve karrikin and plant hormones pathways will not be referred to here; an extensive review of Blázquez and coworkers provides ample and adequate information [66].

## 6. Summing up Perspectives

Doubtless, the discovery and identification of the enormous biological activity of SW and KAR_1_ isolated from plant-derived smoke or synthesized potentially allow use in horticulture and agriculture to restore natural vegetation and weed control strategies. SW- and KAR_1_-priming, like in other types of seed priming, offer potential cost-effective techniques to improve seed germination, seedling emergence and development and enhance stand establishment and yields under non-stressful and stressful conditions. However, data on the impact of SW- and KAR_1_-priming of seeds on plant growth and development in various unfavorable environmental conditions are insufficient. In individual plant species, it is necessary to determine whether the seeds should be sown immediately after priming, whether they could be dried to appropriate water content, and how (fast or slow) they should be dried. It seems that it would be worthwhile to combine SW- and/or KAR_1_-priming with osmopriming, matripriming, or hormopriming, as well as biopriming. In addition to increasing the plant’s tolerance to adverse environmental conditions, priming can also remove seed dormancy. At present, there is some knowledge on dormancy regulation by KAR_1_ in association with the phytohormones ABA and GA_s_ in *Arabidopsis* seeds [17] and *Avena fatua* caryopses [9] as well as in relation to ethylene and regulation of oxidative homeostasis in *A. fatua* caryopses [9,67]. However, information regarding the contribution of plant hormones to the beneficial response of seeds to KAR_1_-priming, from the standpoint of improving seed quality and increasing seed tolerance to suboptimal conditions, is insufficient. 

Finally, the commercial use of both SW and KAR_1_ in seed conditioning and in in vitro appears now to be more cost-effective, compared to stimulation of seed germination in the soil bank or to improving plant development by watering or spraying, as it is associated with lower consumption of both SW and KAR_1_.

## Figures and Tables

**Table 1:** Substrates used for KAR_1_ synthesis.

Substrates	Structure	References
***D-xylose***	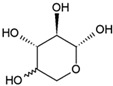	[20,21]
***Ethyl-4-methyl-2-oxo-2,5-dihydro-furan-3-carboxylate***	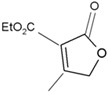	[22]
***Furfurylmethanol***	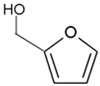	[23]
***Pyromeconic acid***	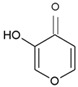	[24]

**Table 2:** Effects of some compounds present in plant-derived smoke on seed germination. ↑stimulation; ↓ inhibition.

Compound	Plant Species		References
Nitrogen dioxide	*Emmenanthe penduliflora*	↑	[26]
KAR_1_ (3-methyl-2*H*-furo [2, 3-c]pyran-2-one)	*Lactuca sativa*	↑	[15,16]
KAR_2_, KAR_3_	*Arabidopsis thaliana*	↑	[17]
KAR_4_	*Lactuca sativa*	↑	[27]
3,4,5-trimethylfuran-2(5*H*)-one (trimethylbutenolide; TMB)	*Lactuca sativa*	↓	[28]
Glyceronitrile	*Angiozanthos manglesi*	↑	[29]
Hydroquinone	*Lactuca sativa*	↑	[30]
5,5-dimethylfuran-2(5*H*) -one	*Lactuca sativa*	↓	[31]
(5RS) -5-ethylfuran-2(5*H*) -one	*Lactuca sativa*	↓	[31]

**Table 3:** Examples of beneficial effects of SW- or KAR_1_-priming.

Plant Species	SW, Dilution KAR_1_, M or μg/L^−1^	Methods of Application	Beneficial Effect on:	References
*Brassica* *napus* L.	1:1000 10^−8^	imbibition at 25 °C for 12 h, blotting dry	final percentage, time of germination, at heat stress at 40 °C, CAT activity after 7 d	[34]
*Brassica* *napus* L.	1:250 10^−7^	imbibition at 25 °C for 12 h, blotting dry	growth of seedlings at 25 °C after 7 d	[34]
*Capsicum annuum* L.	10^−7^	imbibition at 25 °C, for 40 h, rinsing, drying at room temp. for 24 h	germination of immature seeds at 25 °C after 10 d, seedling emergence from immature seeds, fresh weight of seedlings from immature and mature seeds at 23 °C after 20 d	[35]
*Ceratotheca triloba* (Bernh.) Hook.	1:500 10^−6^	imbibition at 25 °C, for 48 h	germination, vigor, seedling growth, at 10/15 °C, 10 °C after 15 d, germination under osmotic stress at 25 °C after 25 d	[36]
*Coriandrum sativum* L.	10^−6^	priming in solution at room temperature for 15 h	seed germination, seedling growth, chlorophyll a, b, carotenoids and proline contents, membrane stability, leaf osmotic potential, photosynthesis rate, the activity of SOD, POD and CAT at 25 °C after 15 d under cadmium stress	[37]
*Cucumis* *melo* L.	10^−7^	imbibition at 25 °C for 21 h, rinsing, surface drying	seedling emergence at 20° and 25 °C, fresh and dry weight of seedling after 24 d	[38]
*Daucus* *carota* L.	51.6 μg/L 1.5 μg/L	soaking for 12 h, rinsing, drying	germination in soil, seedling growth at environmental conditions, photosynthesis, ascorbic acid content after 120 d	[39]
*Eragrostis tef* (Zucc.) Trotter	1:500 10^−8^	imbibition for 48 h at 25 °C	vigor at 20°to 40 °C, seedling length and vigor index, germination at 25–40 °C, at 25 °C under osmotic stress	[40]
*Lycopersicon esculentum* Mill.	10^−7^	soaking at 23 °C for 24 h, drying up to the initial weight	rate of germination, vigor seedling in water at 10°–35 °C, in salt solutions or PEG solutions at 23 °C after seven days	[41]
*Oryza sativa* L.	1:1000 1:500	primed in solutens for 24 h, air drying at room temperature	seed germination up to 3 d under salt stress simulated by NaCl at 30 °C, chlorophyll, carotenoids, K^+^, Ca ^+^ contents	[42]
*Silybum marianum* L. Gaertn. *Solanum centrale* J.M.Black, *S.dioicum* W.Fitzg, *S. orbiculatum* Dunal ex Poir	1:250 1:10 0.67μM	soaking 1 h at room temperature, drying, soaking for 24 h, rinsing	speed of germination, vigor, at 25 °C index seedling length after 14 d germination at 26/13 °C, 33/18 °C on water agar, vigor index seedling length after 18 d	[43] [44]
*Themeda triandra* Forssk.	SW	soaking 1 h at room temperature, drying, and stored at 25 for 3 to 21 days	germination at 25 °C	[45]
*Zea mays* L.	1:500	soaking 6–18 h	seedling growth at 28 °C after 8 d, chlorophyll and carotenoids contents	[46]
*Zea mays* L.	1:500 10^−7^	soaking 1 h, surface drying	seedling growth in the soil after 30 d in greenhouse conditions	[47]

**Table 4:** Effect of SW and KAR_1_ in tissue cultures. + stimulation; − no effect; ND- no data.

Process	Plant Species	SW	KAR_1_	References
Somatic embryogenesis Efficiency	*Baloskion tetraphyllum* *Pelargonium hortorum* *Pinus wallichiana*	ND + +	+ ND ND	[53] [54] [55]
Somatic embryo germination	*Baloskion tetraphyllum* *Pinus wallichiana*	ND +	+ ND	[53] [55]
Conversion to plantlets	*Baloskion tetraphyllum* *Brassica napus* *Pinus wallichiana*	ND + +	+ ND ND	[53] [56] [55]
Secondary embryogenesis	*Brassica napus*	+	ND	[57]
Seed germination	*Ansellia africana* *Baloskion tetraphyllum* *Vanda parviflora* *Xenikophyton smeeanum*	+ ND + +	− + ND ND	[58] [53] [59] [60]

## References

[1] Jisha K.C., Vijayakumari K., Puthur J.T. (2013). Seed priming for abiotic stress tolerance: An overview. Acta Physiol. Plant.

[2] Garcia D., Zhao S., Arif S., Zhao Y., Ming L.C., Huang D. (2022). Seed Priming technology as a key strategy to increase crop plant production under adverse environmental conditions. J. Agri. Horti. Res..

[3] Kępczyński J. (2021). Gas-priming as a novel simple method of seed treatment with ethylene, hydrogen cyanide or nitric oxide. Acta Physiol. Plant.

[4] Farooq M., Usman M., Nadeem F., ur Rehman H., Wahid A., Basra S.M., Siddique K.H. (2019). Seed priming in field crops: Potential benefits, adoption and challenges. Crop Pasture Sci..

[5] Kulkarni M.G., Light M.E., Van Staden J. (2011). Plant-derived smoke: Old technology with possibilities for economic applications in agriculture and horticulture. S. Afr. J. Bot..

[6] De Lange J.H., Boucher C. (1990). Auto ecological studies on *Audinia capitata* (Bruniaceae). I. Plant-derived smoke as a germination cue. S. Afr. J. Bot..

[7] Dixon K.W., Merritt D.J., Flematti G.R., Ghisalberti E.L. (2009). Karrikinolide–a phytoreactive compound derived from smoke with applications in horticulture, ecological restoration and agriculture. Acta Hort..

[8] Nelson D.C., Flematti G.R., Ghisalberti E.L., Dixon K.W., Smith S.M. (2012). Regulation of seed germination and seedling growth by chemical signals from burning vegetation. Ann. Rev. Plant Biol..

[9] Kępczyński J. (2018). Induction of agricultural weed seed germination by smoke and smoke-derived karrikin (KAR 1), with a particular reference to *Avena fatua* L. Acta Physiol. Plant.

[10] Kępczyński J. (2020). Progress in utilizing plant-derived smoke water and smoke-derived KAR1 in plants tissue culture. Plant Cell Tiss. Organ Cult..

[11] Banerjee A., Tripathi D.K., Roychoudhury A. (2019). The Karrikin ‘calisthenics’: Can compounds derived from smoke help in stress tolerance?. Physiol. Plant.

[12] Soós V., Badics E., Incze N., Balázs E. (2019). Fire-borne life: A brief review of smoke-induced germination. Nat. Prod. Commun..

[13] Brown N.A.C., Van Staden J. (1997). Smoke as a germination cue: A review. Plant Growth Regul..

[14] Light M.E., van Staden J. (2004). The potential of smoke in seed technology. S. Afr. J. Bot..

[15] Van Staden J., Jager A.K., Light M.E., Burger B.V. (2004). Isolation of the major germination cue from plant-derived smoke. S. Afr. J. B.

[16] Flematti G.R., Ghisalberti E.L., Dixon K.W., Trengove R.D. (2004). A compound from smoke that promotes seed germination. Science.

[17] Nelson D.C., Riseborough J.A., Flematti G.R., Stevens J., Ghisalberti E.L., Dixon K.W., Smith S.M. (2009). Karrikins discovered in smoke trigger Arabidopsis seed germination by a mechanism requiring gibberellic acid synthesis and light. Plant Physiol..

[18] Nelson D.C., Scaffidi A., Dun E.A., Waters M.T., Flematti G.R., Dixon K.W., Beveridge C.A., Ghisalberti E.L., Smith S.M. (2011). F-box protein MAX2 has dual roles in karrikin and strigolactone signaling in *Arabidopsis thaliana*. Proc. Nat. Acad. Sci. USA.

[19] Kępczyński J., Cembrowska D., Van Staden J. (2010). Releasing primary dormancy in *Avena fatua* L. caryopses by smoke-derived butanolide. Plant Growth. Regul..

[20] Goddard-Borger E.D., Ghisalberti E.L., Stick R.V. (2007). Synthesis of the Germination Stimulant 3-Methyl-2H-furo [2, 3-c] pyran-2-one and Analogous Compounds from Carbohydrates. Eur. J. Org. Chem..

[21] Matsuo K., Shindo M. (2011). Efficient synthesis of karrikinolide via Cu (II)-catalyzed lactonization. Tetrahedron.

[22] Sun K., Chen Y., Wagerle T., Linnstaedt D., Currie M., Chmura P., Xu M. (2008). Synthesis of butenolides as seed germination stimulants. Tetrahedron Lett..

[23] Nagase R., Katayama M., Mura H., Matsuo N., Tanabe Y. (2008). Synthesis of the seed germination stimulant 3-methyl-2H-furo [2, 3-c] pyran-2-ones utilizing direct and regioselective Ti-crossed aldol addition. Tetrahedron Lett..

[24] Flematti G.R., Gavin R., Ghisalberti E.L., Dixon K.W., Trengove R.D. (2005). Synthesis of the seed germination stimulant 3-methyl-2H-furo[2, 3-c]pyran-2-one. Tetrahedron Lett..

[25] Flematti G.R., Waters M.T., Scaffidi A., Merritt D.J., Ghisalberti E.L., Dixon K.W., Smith S.M. (2013). Karrikin and cyanohydrin smoke signals provide clues to new endogenous plant signaling compounds. Mol. Plant..

[26] Keeley J.E., Fotheringham C.J. (1997). Trace gas emissions in smoke-induced germination. Science.

[27] Flematti G.R., Goddard-Borger E.D., Merritt D.J., Ghisalberti E.L., Dixon K.W., Trengove R.D. (2007). Preparation of 2 H-furo [2,3-c] pyran-2-one derivatives and evaluation of their germination-promoting activity. J. Agr. Food Chem..

[28] Light M.E., Burger B.V., Staerk D., Kohout L., van Staden J. (2010). Butenolides from plant-derived smoke: Natural plant-growth regulators with antagonistic actions on seed germination. J. Nat. Prod..

[29] Flematti G.R., Merritt D.J., Piggott M.J., Trengove R.D., Smith S.M., Dixon K.W., Ghisalberti E.L. (2011). Burning vegetation produces cyanohydrins that liberate cyanide and stimulate seed germination. Nat. Commun..

[30] Kamran M., Khan A.L., Ali L., Hussain J., Waqas M., Al-Harrasi A., Imran Q.M., Kim Y.H., Kang S.M., Yun B.W. (2017). Hydroquinone: A novel bioactive compopund from plant-derived smoke can cue seed germination of lettuce. Front. Chem..

[31] Burger B.V., Pošta M., Light M.E., Kulkarni M.G., Viviers M.Z., Van Staden J. (2018). More butenolides from plant-derived smoke with germination inhibitory activity against karrikinolide. S. Afr. J. Bot..

[32] Light M.E., Gardner M.J., Jager A.K., van Staden J. (2002). Dual regulation of seed germination by smoke solutions. Plant Growth Regul..

[33] Khatoon A., Rehman S.F., Aslam M.M., Jamil M., Komatsu S. (2020). Plant-derived smoke affects biochemical mechanism on plant growth and seed germination. Inter. J. Mol. Sci..

[34] Moyo M., Amoo S.O., Van Staden J. (2022). Seed priming with smoke water and karrikin improves germination and seedling vigor of Brassica napus under varying environmental conditions. Plant Growth Regul..

[35] Demir I., Ozden E., Yıldırım K.C., Sahin O., Van Staden J. (2018). Priming with smoke-derived karrikinolide enhances germination and transplant quality of immature and mature pepper seed lots. S. Afr. J. Bot..

[36] Masondo N.A., Kulkarni M.G., Finnie J.F., Van Staden J. (2018). Influence of biostimulants-seed-priming on Ceratotheca triloba germination and seedling growth under low temperatures, low osmotic potential and salinity stress. Ecotoxicol. Environ. Saf..

[37] Sardar R., Ahmed S., Yasin N.A. (2021). Seed priming with karrikinolide improves growth and physiochemical features of Coriandrum sativum under cadmium stress. Environ. Adv..

[38] Mavi K., Light M.E., Demir I., Van Staden J., Yasar F. (2010). Positive effect of smoke-derived butenolide priming on melon seedling emergence and growth. N. Z. J. Crop Hort. Sci..

[39] Akeel A., Khan M.M.A., Jaleel H., Uddin M. (2019). Smoke-saturated Water and Karrikinolide Modulate Germination, Growth, Photosynthesis and Nutritional Values of Carrot (*Daucus carota* L.). J. Plant Growth Regul..

[40] Ghebrehiwot H.M., Kulkarni M.G., Kirkman K.P., Van Staden J. (2008). Smoke-water and a smoke-isolated butenolide improve germination and seedling vigour of Eragrostis Tef (Zucc.) Trotter under high temperature and low osmotic potential. J. Agron. Crop Sci..

[41] Jain N., Van Staden J. (2007). The potential of the smoke-derived compound 3-methyl-2H-furo[2, 3-c]pyran-2-one as a priming agent for tomato seeds. Seed Sci. Res..

[42] Jamil M., Kanwal M.A., Aslam M.M., Khan S., Tu J., Réhman S.U. (2014). Effect of plant-derived smoke priming on physiological and biochemical characteristics of rice under salt stress condition. Austr. J. Crop Sci..

[43] Abdollahi M.R., Mehrshad B., Moosavi S.S. (2011). Effect of method of seed treatment with plant derived smoke solutions on germination and seedling growth of milk thistle (*Silybum marianum* L.). Seed Sci. Technol..

[44] Commander L.E., Merritt D.J., Rokich D.P., Flematti G.R., Dixon K.W. (2008). Seed germination of *Solanum* spp. (Solanaceae) for use in rehabilitation and commercial industries. Aust. J. Bot..

[45] Baxter B.J.M., Van Staden J. (1994). Plant-derived smoke: An effective seed pre-treatment. Plant Growth Regul..

[46] Aslam M.M., Jamil M., Khatoon A., Hendawy S.E., Al-Suhaibani N.A., Malook I., Rehman S.U. (2017). Physiological and biochemical responses of maize (*Zea mays* L.) to plant derived smoke solution. Pak. J. Bot..

[47] Van Staden J., Sparg S.G., Kulkarni M.G., Light M.E. (2006). Post-germination effects of the smoke-derived compound 3-methyl-2H-furo [2,3-c] pyran-2-one, and its potential as a preconditioning agent. Field Crops Res..

[48] Abu Y., Romo J.T., Bai Y., Coulman B. (2016). Priming seeds in aqueous smoke solution to improve seed germination and biomass production of perennial forage species. Can. J. Plant Sci..

[49] Keeley J.E. (1993). Smoke-induced flowering in the fire-lily Cyrtanthus ventricosus. S. Afr. J. Bot..

[50] Kulkarni M.G., Ascough G.D., Van Staden J. (2007). Improved flowering of a South African Watsonia with smoke treatment. S. Afr. J. Bot..

[51] Papenfus H.B., Kumari A., Kulkarni M.G., Finnie J.F., Van Staden J. (2014). Smoke-water enhances in vitro pollen germination and tube elongation of three species of Amaryllidaceae. S. Afr. J. Bot..

[52] Kumari A., Papenfus H.B., Kulkrni M.G., Pošta M., van Staden J. (2015). Efect of smoke derivatives on in vitro pollen germination and pollen tube elongation of species from diferent plant families. Plant Biol..

[53] Ma G.-H., Bunn E., Dixon K., Flematti G. (2006). Comparative enhancement of germination and vigor in seed and somatic embryos by the smoke chemical 3-methyl-2H-furo [2,3-C] pyran-2-one in *Baloskion tetraphyllum* (Restionaceae). Vitr. Cell Dev. Biol..

[54] Senaratna T., Dixon K., Bunn E., Touchell D. (1999). Smoke-saturated water promotes somatic embryogenesis in geranium. Plant Growth Regul..

[55] Malabadi R.B., Nataraja K. (2007). Smoke-saturated water infuences somatic embryogenesis using vegetative shoot apices of mature trees of Pinus wallichiana AB Jacks. J Plant Sci..

[56] Ghazanfari P., Abdollahi M.R., Moieni A., Moosavi S.S. (2012). Efect of plant-derived smoke extract on in vitro plantlet regeneration from rapeseed (*Brassica napus* L. cv. *Topas*) microspore-derived embryos. Int. J. Plant Prod..

[57] Abdollahi M.R., Ghazanfari P., Corral-Martínez P., Moieni A., Seguí-Simarro J.M. (2012). Enhancing secondary embryogenesis in Brassica napus by selecting hypocotyl- derived embryos and using plant-derived smoke extract in culture medium. Plant Cell Tissue Organ Cult..

[58] Papenfus H.B., Naidoo D., Pošta M., Finnie J.F., Van Staden J. (2016). The efects of smoke derivatives on in vitro seed germination and development of the leopard orchid Ansellia africana. Plant Biol..

[59] Malabadi R.B., da Silva J.A.T., Mulgund G.S. (2008). Smoke-saturated water influences in vitro seed germination of Vanda parviflora Lindl. Seed Sci. Biotechnol..

[60] Malabadi R.B., Vijaykumar S., da Silva J.A.T., Mulgund G.S., Nataraja K. (2011). In vitro seed germination of an epiphytic orchid Xenikophyton smeeanum (Reichb. f.) by using smoke-saturated-water as a natural growth promoter. Int. J. Biol. Technol..

[61] Espinosa-Leal C.A., Garcia-Lara S. (2020). In vitro germination and initial seedling development of krantz aloe by smoke-saturated water and seed imbibition. Hort Technol..

[62] Monthony A.S., Baethke K., Erland L.A., Murch S.J. (2020). Tools for conservation of Balsamorhiza deltoidea and Balsamorhiza sagittata: Karrikin and thidiazuron-induced growth. Vitr. Cell. Develop. Biol.-Plant..

[63] Waters M.T., Nelson D.C. (2023). Karrikin perception and signalling. New Phytol..

[64] Morffy N., Faure L., Nelson D. (2016). Smoke and hormone mirrors:action and evolution of Karrikin and strigolactone signaling. Trends Genet..

[65] Bürger M., Mashiguchi K., Lee H.J., Nakano M., Takemoto K., Seto Y., Yamaguchi S., Chory J. (2019). Structural. basis of Karrikin and non-natural strigolactone perception in *Physcomitrella patens*. Cell Rep..

[66] Blázquez M.A., Nelson D.C., Weijers D. (2020). Evolution of plant hormone response pathways. Annu. Rev. Plant. Biol..

[67] Kępczyński J., Soumya M., Aftab T. (2023). Induction of dormancy release in agricultural weed seeds by plant-derived smoke and smoke-derived Karrikin 1(KAR1). A relationship with plant hormones. Strigolactones, Karrikins and Alkamides in Plants.

